# Biological Interplay between Orthodontic Forces and Apical Papilla-derived Stem Cells: A Review on Mechanisms and Implications -

**DOI:** 10.31661/gmj.vi.3946

**Published:** 2025-11-08

**Authors:** Maryam Sobhani, Yasaman Bathaei, Alireza Afzalan, Sepideh Hormozi, Zeinab Shanehsaz, Sara Nabizadeh, Kosar Gashtasb

**Affiliations:** ^1^ Department of Orthodontics, School of Dentistry, Tehran University of Medical Sciences, Tehran, Iran; ^2^ Department of Oral and Maxillofacial Surgery of Ahvaz Jundishapur University of Medical Sciences, Ahvaz, Iran; ^3^ Department of Orthodontics, School of Dentistry, Ahvaz Jundishapur University of Medical Sciences, Ahvaz, Iran; ^4^ Department of Periodontics, Faculty of Dentistry, Shahid Sadoughi University of Medical Sciences, Yazd, Iran; ^5^ Department of Restorative Dentistry, School of Dentistry, Tehran University of Medical Sciences, Tehran, Iran

**Keywords:** Biological Interplay, Orthodontic Forces, Apical Papilla

## Abstract

**Background:**

Orthodontics is not limited to the movement of teeth, but has also expanded to the cellular and molecular realms. In this study, it was aimed to review mechanisms and implications of biological interplay between orthodontic forces and apical papilla-derived stem cells.

**Materials and Methods:**

This narrative and semi-systematic review investigates the biological interactions between orthodontic forces and stem cells derived from the apical papilla (SCAPs). A comprehensive literature search was conducted in PubMed, Web of Science, and Google Scholar using specific keywords and MeSH terms from 2015 to 2025. Studies in English and Persian were included if they focused on the effects of orthodontic forces on SCAPs’ function, differentiation, or signaling pathways. Key biological mechanisms and molecular responses were extracted and analyzed.

**Results:**

Between 2015 and 2025, 10 studies were included, primarily investigating human dental stem cells (SCAPs, DPCs, PDLSCs, SHEDs) and rat models. Findings showed that advanced glycation end-products inhibit osteogenesis via KDM6B and Wnt/β-catenin suppression, whereas Ape1 inhibition promotes odontogenic differentiation through the same pathway. Nano-dentine enhanced osteogenic gene expression compared to MTA and Biodentine, and low-energy blue LED stimulated osteogenic differentiation despite reduced proliferation. TGF-β1 had dose-dependent effects on SCAP proliferation and differentiation, while FTO/SMOC2 regulated odontoblastic differentiation under inflammatory conditions. Mechanical forces reduced SHED proliferation without affecting apoptosis, and pathogenic bacteria like F. nucleatum and E. faecalis impaired SCAP proliferation, viability, and osteogenic gene expression, indicating that molecular, material, mechanical, and microbial factors critically modulate dental stem cell differentiation.

**Conclusion:**

Current evidence shows that various physical, chemical, microbial, and molecular factors influence dental stem cell behavior. Understanding these mechanisms can support the development of personalized therapies, enhancing outcomes in endodontic and orthodontic treatments, particularly under pathological conditions like diabetes and chronic inflammation.

## Introduction

Orthodontics is not a concept that is limited to the movement of teeth, but has also expanded to the cellular and molecular realms. While the primary goals of orthodontic treatment include improving function, aesthetics, and dental health, the application of mechanical forces to teeth induces a complex set of biological responses in the surrounding tissues, including the periodontal ligament, cementum, dental pulp, and alveolar bone. A less studied but very important aspect of this field is the effect of orthodontic forces on stem cells present in developing teeth, particularly stem cells derived from the apical papilla (SCAP) [1].

The apical papilla is located in the apical region of immature permanent teeth and contains a mesenchymal tissue containing stem cells. SCAP cells have been identified as a subset of mesenchymal stem cells (MSCs) that can differentiate into osteoblasts, adipocytes, chondrocytes, and odontoblast-like cells [2]. The characteristics of these cells, such as high proliferative potential, broad differentiation capacity, and the ability to secrete growth factors, make them valuable resources for pulp tissue engineering and root regeneration [3]. Because most orthodontic treatments are performed during adolescence, when the roots of many teeth are not yet fully developed, the interaction between orthodontic mechanical forces and SCAP can have important effects on the process of root formation and pulp health.

Mechanical stimuli, such as pressure, tension, and vibration, on stem cells can lead to the activation of diverse signaling pathways that ultimately alter cellular behavior such as differentiation, migration, apoptosis, or cytokine secretion [4].

In general, SCAPs are sensitive to mechanical stimuli and can alter the expression of specific genes, such as Runx2, ALP, OCN, and other osteogenic markers, and act as a driver in these cells [5]. The result of these responses is that they promote root formation, strengthen alveolar bone, and even prevent or exacerbate root resorption.

Furthermore, excessive or improper forces can lead to disruption of SCAP activity and the weakening of the normal differentiation processes of these cells. Some studies have shown that high mechanical stress can lead to increased oxidative stress and induction of apoptosis in SCAP [6]. This observation is of great importance because it can lead to the arrest or slowdown of tooth root growth, or even irreversible pulp damage.

Evaluation of the interaction of orthodontic forces with SCAP can also have a significant impact on the design of new treatment strategies.

It is of particular interest that in the future; by controlling the type and intensity of orthodontic forces, it may be possible to stimulate desirable biological processes such as enhancing bone formation, accelerating root growth, or reducing root resorption, leading to significant advances in science. Also, in combination with tissue regeneration therapies, such as the use of biomaterials or growth factors, this knowledge can lead to the design of individualized treatments for adolescent patients [7].

Also, if the signaling pathways involved, such as the Wnt/β-catenin, ERK1/2, PI3K/Akt, and TGF-β/Smad pathways, are understood in greater detail, it could open up new opportunities for pharmacological or genetic intervention to improve treatment outcomes [8]. However, many of the existing studies are limited to in vitro conditions, and further research in in vivo settings and animal models is needed to confirm these findings. Therefore, in this study, it was aimed to review mechanisms and implications of biological interplay between orthodontic forces and apical papilla-derived stem cells.

## Materials and Methods

### Study Design

This study is a narrative and semi-systematic review following the Preferred Reporting Items for Systematic Reviews and Meta-Analyses (PRISMA) guidelines. The review aimed to evaluate the biological effects of orthodontic forces on stem cells derived from the apical papilla (SCAPs), focusing on molecular, cellular, and clinical mechanisms underlying osteogenic and odontogenic differentiation (Table-1).

### Data Sources and Search Strategy

A comprehensive literature search was performed in PubMed, Web of Science, and Google Scholar. Search terms included a combination of keywords and MeSH terms: "apical papilla stem cells", "stem cells from apical papilla (SCAPs)", "orthodontic force", "mechanotransduction", "dental stem cells", "osteogenic differentiation", "periodontal remodeling", and "mechanobiology in orthodontics". The search covered publications from January 2015 to 2025. Only full-text articles published in English and Persian were included. Articles without full access or irrelevant to the study objectives were excluded.

### Inclusion and Exclusion Criteria

Studies were included if they met the following criteria:

• Investigated the effects of orthodontic forces or mechanical stimuli on SCAPs;

• Explored biological mechanisms, signaling pathways, or gene responses;

• Were peer-reviewed in vitro, in vivo, or clinical studies.

Exclusion criteria were:

• Studies unrelated to SCAPs or orthodontic forces;

• Studies with incomplete methodology or insufficient data;

• Non-peer-reviewed articles, reviews, or conference abstracts.

### Study Selection

The selection process followed PRISMA guidelines. Two independent reviewers screened titles and abstracts for relevance. Disagreements were resolved by a third reviewer. Full texts of eligible studies were then assessed, and key data were extracted, including study design, sample size, model type, interventions, and main outcomes.

### Data Extraction and Synthesis

From each included study, data were collected on:

• Study type (in vitro, in vivo, or combined);

• Sample characteristics (human SCAPs, SHEDs, DPCs, PDLSCs, or animal models);

• Interventions (mechanical force, biomaterials, LED exposure, gene modulation, bacterial challenge, pharmacological treatments);

• Outcomes measured (osteogenic/odontogenic differentiation, proliferation, mineralization, cytokine expression).

The extracted data were summarized descriptively, highlighting the molecular and cellular mechanisms by which orthodontic forces influence SCAP behavior.

### Risk of Bias Assessment

The risk of bias was evaluated using the PRILE checklist for dental in-vitro studies [9], considering study design, sample size, randomization, blinding, outcome measurement, statistical methods, and generalizability. Each domain was categorized as low, moderate, or high risk.

## Results

**Table:** Table1. characteristics of included studies

**Study ID**	**Study Design**	**Sample (n)**	**Models Used**	**Key Interventions / Groups**	**Main Methods**
Ying et al., 2024	In vivo and in vitro experimental	Rats (n=5/group); Human PDLSCs (n=5 donors)	OTM rat model, T2DM rat model, human PDLSCs	Control, OTM, T2DM+OTM+nT, T2DM+OTM+FPS-ZM1, OTM+GSK-J4	Bioinformatics (GSE112122, GSE1946), RNA-seq, qRT-PCR, Western blot, ELISA, IHC, IF, ALP/ARS staining, mechanical loading, inhibitors (FPS-ZM1, GSK-J4, LiCl, XAV939, KN-93), ROS detection
Saberi et al., 2022	In vitro experimental (cell-material interaction)	SCAPs (n=3 donors), 36 dentin blocks	Human SCAPs cultured with dentine cement blocks	Nano-dentine, MTA, Biodentine	Cement synthesis via sol-gel; SEM, XRD, FTIR, biodegradation, MTS assay, Alizarin Red staining, qRT-PCR for COL1A1, RUNX2, SPP1, TGFB1, iNOS
Yang et al., 2020	In vitro experimental	SCAPs from 3 donors	Human SCAPs exposed to blue LED	0 (control), 1, 2, 3, 4 J/cm² blue LED	Cell isolation and culture; MTT assay; ALP staining (7-14 days); Alizarin Red mineralization (28 days); qRT-PCR for ALP, DSPP, DMP1, OCN
Chen et al., 2015	In vitro and in vivo experimental	Human DPCs (n=12 donors); Rat tooth germs	Human dental papilla cells; Rat tooth development model	Control, Ape1-shRNA, E3330, E3330+DKK1	Immunohistochemistry (rat tooth germs), DPC isolation, shRNA knockdown, E3330 treatment, ALP/ARS assays, qRT-PCR, Western blot, flow cytometry, DKK1 inhibition, cell cycle assays
Chang et al., 2015	In vitro experimental	Human SCAPs (3 strains, 8-9 years old)	Human SCAP culture exposed to TGF-β1	Control, TGF-β1 (0.1-10 ng/mL), ±SB431542, ±U0126	MTT assay, collagen assay (Sircol), ALP activity, Western blot for p-Smad2 and p-ERK1/2, inhibitor pretreatments (SB431542 for ALK5/Smad2/3; U0126 for MEK/ERK)
Ebadi et al., 2023	In vitro experimental	Human SCAPs from third molars (n not specified)	Human SCAPs cultured in differentiation microenvironment	Control, differentiation microenvironment for 21 days	SCAP isolation and culture (DMEM + 10% FBS + penicillin/streptomycin), flow cytometry for CD90, CD73, CD34, differentiation in DMEM + 10% FBS + ß-glycerophosphate + ascorbic acid for 21 days, ALP assay, Alizarin Red S staining for calcium deposition, qRT-PCR for CEMP1, COL1, SPON1, OCN, OPN at days 7, 14, 21, statistical analysis via one-way ANOVA
Huang et al., 2023	In vivo (AP rat model) + in vitro (hSCAPs)	16 SD rats; hSCAPs from 16-18 y.o. donors	AP rat model; human SCAPs cultured in odontoblastic induction medium (OM) ± LPS	Control, LPS, OM, OM+LPS; FTO overexpression/knockdown; SMOC2 knockdown	Micro-CT, HE, IF staining of roots; hSCAP isolation and characterization; ALP & ARS staining; qRT-PCR & Western blot for DSPP, DMP1, COL1, FTO, SMOC2; siRNA & lentiviral transfection for FTO/SMOC2; mRNA stability assay
Liu et al., 2022	In vitro experimental	SHEDs isolated from deciduous teeth	SHEDs cultured under mechanical centrifugal force (0, 100, 200, 300 g for 30 min/day, 7 days)	Control (0 g), mechanical forces (100, 200, 300 g)	Cell proliferation assay (CCK-8), flow cytometry for cell cycle & apoptosis, TEM for morphology
Rakhimova et al., 2025	In vitro experimental	Human SCAPs (n=3 donors)	SCAPs exposed to oral bacteria under anaerobic conditions	F. nucleatum, E. faecalis, A. gerensceriae, S. exigua, L. gasseri, L. reuteri	SCAP isolation and culture; flow cytometry; confocal fluorescence microscopy; xCELLigence real-time proliferation assay; trypan blue viability assay; cytokine multiplex assays (IL-6, IL-8, IL-10, TGF-β1/2/3); pH measurement; statistical analyses (ANOVA, Wilcoxon, Spearman correlation)
Razghonova et al., 2022	In vitro transcriptomic	Human SCAPs from 3 donors	SCAP culture	Control, F. nucleatum (planktonic), F. nucleatum supernatant, E. faecalis (planktonic), E. faecalis supernatant	SCAP isolation and characterization; co-culture with bacteria/supernatants (MOI 100) for 24 h; RNA extraction; RNA-seq; DEG analysis; GO enrichment; Tanimoto coefficient for similarity; PCA

**Figure-1:**
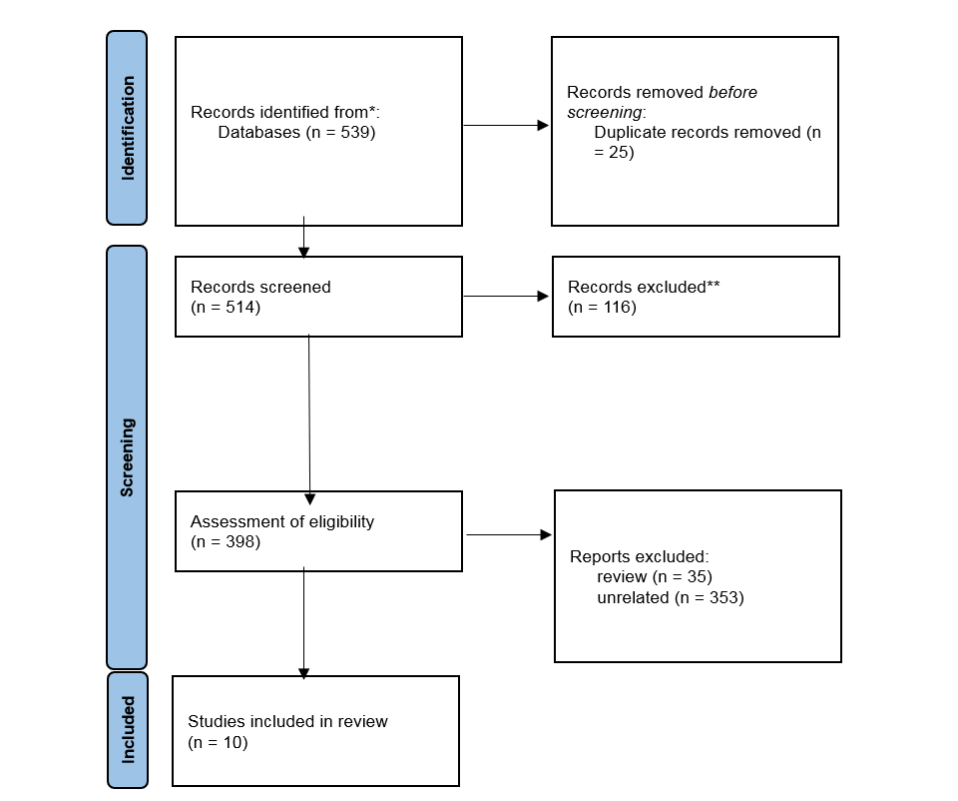
Figure 1. PRISMA Flowchart for selecting of the literature related to this review

**Table:** Table2. Risk of Bias based on the PRILE Checklist for Individual Studies

	Ying et al., 2024	Nano-Dentine & SCAPs Study	Yang et al., 2020	Chen et al., 2015	Chang et al., 2015	Ebadi et al., 2023	Huang et al., 2023	Liu et al., 2022	Rakhimova et al., 2025	Razghonova et al., 2022
Study design	Low	Low	Low	Low	Low	Moderate	Moderate	Moderate	Low	Low
Sample size	Low	Low	Low	Low	Moderate	Low	Low	Low	Low	Low
Inclusion/exclusion criteria	Low	Low	Low	Low	Low	Low	Low	Moderate	Low	Low
Randomisation	Moderate	Moderate	Moderate	Moderate	Moderate	High	Moderate	Low	Moderate	Moderate
Blinding	Moderate	Moderate	Moderate	Moderate	Moderate	High	High	Low	Moderate	Moderate
Outcome measures	Low	Low	Low	Low	Low	Low	Low	Low	Low	Low
Statistical methods	Low	Low	Low	Low	Low	Low	Low	Low	Low	Low
Experimental animals/cells	Low	Low	Low	Low	Low	Low	Low	Low	Low	Low
Experimental procedures	Low	Low	Low	Low	Low	Low	Low	Low	Low	Low
Results	Low	Low	Low	Low	Low	Low	Low	Low	Low	Low
Discussion/interpretation	Low	Low	Low	Low	Low	Low	Low	Low	Low	Low
Generalisability/translation	Moderate	Moderate	Moderate	Moderate	Moderate	Moderate	Moderate	Moderate	Moderate	Moderate

Between 2015 and 2025, 539 records were identified. Based on the search keywords 398, studies were remined from records screened, and finally, based on Figure-1, 10 records were included in the study [10][11][12][13][14][15][16][17][18][19].

Of the 10 included studies, 7 were in vitro experimental and 3 combined in vitro and in vivo approaches. Of the 10 included studies, 7 (70%) used human dental stem cells (SCAPs, DPCs, PDLSCs, or SHEDs) with donor numbers ranging from 3 to 12, 3 (30%) included rat samples (5-16 animals per group), and 1 (10%) used 36 dentin blocks for in vitro experiments. Of the 10 included studies, 7 (70%) used human dental stem cell models (SCAPs, PDLSCs, DPCs, or SHEDs) under various in vitro conditions, 3 (30%) used rat models (OTM, T2DM, or AP), and several studies combined human cells with experimental manipulations such as LED exposure, TGF-β1, LPS, mechanical force, or bacterial challenge. Included studies investigated a range of interventions and experimental groups, including disease and pharmacological models (e.g., OTM, T2DM+OTM with FPS-ZM1 or GSK-J4 in rats), material comparisons (nano-dentine, MTA, Biodentine), varying doses of blue LED, gene modulation (Ape1-shRNA, E3330, TGF-β1, SMOC2/FTO knockdown or overexpression), differentiation microenvironments, mechanical force exposures (0-300 g), and bacterial challenges with multiple species or bacterial supernatants. Studies employed a wide array of methods, including bioinformatics and RNA-seq analyses, cell isolation and culture, gene modulation (shRNA, siRNA, lentiviral transfection), immunohistochemistry, Western blot, qRT-PCR, ALP and Alizarin Red staining, flow cytometry, mechanical loading, pharmacological inhibitors, micro-CT, TEM, confocal microscopy, cytokine assays, co-culture with bacteria, cement synthesis and characterization (SEM, XRD, FTIR), and various statistical analyses to evaluate proliferation, differentiation, mineralization, and molecular signaling. Of the total studies, 8 focused on stem cells derived from the apical papilla (SCAPs), which were mostly cultured in vitro. Most of the studies investigated timescales between 7 and 21 days for induction of differentiation. In terms of microbial conditions, two important studies [18][19] specifically investigated the effects of root pathogenic bacteria, including Fusobacterium nucleatum and Enterococcus faecalis. The countries where the studies were conducted mainly included China, Iran, USA, India, Thailand and Russia, which indicates the geographical spread of the subject and its importance at the international level as shown in Table-1.

The included studies investigate various molecular and cellular mechanisms influencing osteogenic and odontogenic differentiation of stem cells from the apical papilla (SCAPs) and stem cells from human exfoliated deciduous teeth (SHEDs). Ying et al. (2024) explored how advanced glycation end-products (AGEs) suppress force-induced osteogenesis through KDM6B inhibition, highlighting the role of the Wnt/β-catenin-Ca²+-SOD2 axis. Saberi et al. (2022) assessed a novel calcium silicate-pectin nano-dentine, finding it non-toxic with enhanced osteogenic gene expression compared to existing materials. Yang et al. (2020) demonstrated that low-energy blue LED light promotes osteogenic differentiation despite reduced proliferation. Chen et al. (2015) showed that inhibiting Ape1 redox function enhances odontogenic differentiation via Wnt/β-catenin signaling. Chang et al. (2015) revealed TGF-β1’s dual role in SCAP proliferation and differentiation through Smad2/3 and ERK pathways. Ebadi et al. (2023) confirmed SCAP differentiation into cementoblasts with increased ALP and calcium deposition. Huang et al. (2023) identified FTO’s role in odontoblastic differentiation under inflammation, mediated by SMOC2. Liu et al. (2022) found mechanical force reduces SHED proliferation without affecting apoptosis. Lastly, Rakhimova et al. (2025) and Razghonova et al. (2022) investigated oral bacteria’s impact on SCAPs, showing reduced proliferation, altered cytokine secretion, and modulated osteogenic gene expression under bacterial influence.

### Evidence Synthesis

The studies collectively highlight the complex interplay of molecular pathways, environmental factors, and material interventions in regulating osteogenic and odontogenic differentiation of SCAPs and SHEDs. Ying et al. (2024) and Chen et al. (2015) underscore the critical role of Wnt/β-catenin signaling in osteogenesis, with AGEs inhibiting this pathway via KDM6B suppression and Ape1 redox inhibition promoting it, suggesting a delicate balance in redox and signaling regulation.

Similarly, Huang et al. (2023) emphasize FTO’s role in odontoblastic differentiation through SMOC2 under inflammatory conditions, indicating that inflammatory microenvironments significantly modulate differentiation outcomes. Saberi et al. (2022) and Ebadi et al. (2023) demonstrate the potential of biomaterials and differentiation protocols to enhance osteo/odontogenic outcomes, with nano-dentine and cementoblast differentiation protocols upregulating key markers like RUNX2, SPP1, CEMP1, and OCN.

Yang et al. (2020) and Chang et al. (2015) reveal external stimuli’s dual effects: low-energy blue LED enhances differentiation despite reduced proliferation, while TGF-β1’s dose-dependent effects on ALP via Smad2/3 and ERK pathways highlight context-specific responses. Liu et al. (2022) suggest mechanical forces primarily affect proliferation rather than apoptosis, indicating physical stimuli’s selective impact. Rakhimova et al. (2025) and Razghonova et al. (2022) show that oral bacteria, particularly E. faecalis and F. nucleatum, disrupt SCAP proliferation and viability while modulating inflammatory and osteogenic gene expression (e.g., IL-8, RUNX2, NFIL3), pointing to microbial influences on regenerative potential.

### Risk of Bias

Overall, most studies demonstrated low risk of bias across domains such as study design, sample size, outcome measures, statistical methods, and experimental procedures. However, moderate to high risk was noted in randomisation and blinding in several studies, indicating potential limitations in internal validity due to factors like blinding.

## Discussion

Several studies have shown that various factors can influence the differentiation and growth of dental stem cells, especially stem cells from apical papilla (SCAPs) and dental mesenchymal stem cells (DMSCs). These factors include microbial conditions, metabolic changes, mechanical stimuli, light, and biocompatible materials, which are reviewed in more detail based on recent studies.

In contrast to our review findings, a related review by Rojasawasthien et al. (2024), focuses on human periodontal ligament stem cells (hPDLSCs) and their inflammatory responses to various mechanical forces, including compressive, shear, and tensile stresses, showing the role of pro-inflammatory cytokines (IL-1β, IL-6, TNF-α) and toll-like receptor 4 (TLR4) signaling in periodontal tissue remodeling [20]. While our semi-systematic review (n=10 studies) provides detailed insights into SCAP-specific mechanisms and their orthodontic applications, particularly under pathological conditions like diabetes, Rojasawasthien et al.’s narrative review explores a broader range of mechanical forces and their impact on hPDLSCs, emphasizing immune modulation and bone remodeling.

In the study by Saberi et al. [10], a novel nano-dentine cement was investigated and found to induce odontogenic/osteogenic differentiation of SCAPs more than MTA and Biodentine without causing cytotoxicity. This cement increased the expression of RUNX2, SPP1, and TGFB1 genes, which play an important role in cell differentiation.

Yang et al. [11] studied the effect of low-energy blue LED light irradiation. The results showed that although SCAPs proliferation was reduced, osteogenic differentiation was enhanced by increasing the expression of ALP, DSPP, and DMP-1; these findings indicate a positive role of light in cell differentiation.

In this regard, Chen et al. [12] showed that inhibition of the redox activity of the Ape1 enzyme by the inhibitor E3330 increased the osteogenic/odontogenic differentiation of DPCs, which is mediated by activation of the Wnt signaling pathway.

Chang et al. [13] showed that the growth factor TGF-β1 can affect the growth, collagen synthesis, and differentiation of SCAPs through the activation of the ALK5/Smad2/3 and MEK/ERK pathways; such that stimulation or inhibition of these pathways plays a decisive role in the response of cells to TGF-β1, and these findings provide a new horizon for the use of SCAP in dental pulp regeneration and apexogenesis.

In addition, the ability of SCAP to respond to mechanical load makes them a potentially valuable cell population for regenerative endodontic treatments. Future research should focus on determining the optimal range of intensity and duration of applied forces to optimize cell function while maintaining cell survival. It also seems necessary to investigate individual and systemic factors such as inflammation or patient age in modulating the response of SCAP to mechanical forces.

According to Ebadi et al. [14], human apical papilla-derived stem cells (hSCAPs) are capable of successfully differentiating into cementoblasts in vitro, with significant increases in alkaline phosphatase activity, calcium deposition, and expression of specific genes such as CEMP1, COL1, and OPN observed at day 21.

In a recent study, Huang et al. [15] showed that FTO protein plays a positive role in odontoblast differentiation of hSCAPs by increasing SMOC2 expression; this effect is attenuated in inflammatory conditions by decreasing FTO expression, while increasing its expression can counteract the inhibitory effects of lipopolysaccharide (LPS).

According to Liu et al. [17], applying mechanical force to deciduous deciduous tooth pulp stem cells (SHEDs) reduces cell proliferation and changes in cell morphology, but does not affect their apoptosis rate; these findings indicate the high sensitivity of SHEDs to physical stimuli in vitro.

Rakhimova et al [18]. showed that contact of stem cells of the apical papilla (SCAPs) with pathogenic bacteria of the tooth root, such as Fusobacterium nucleatum and Enterococcus faecalis, reduces cell proliferation and changes in the secretion of cytokines, such as IL-8, IL-10, and TGF-β.

This inflammatory response and decreased cellular function indicate the sensitivity of SCAPs to the microbial conditions of the hypoxic environment of the infected pulp. On the other hand, probiotic strains, such as Lactobacillus gasseri and Lactobacillus reuteri, did not have such effects. These findings highlight the importance of considering the microbial conditions of the root environment in the design of regenerative therapies using SCAPs.

Razghonova et al [19]. used transcriptome analysis to show that Fusobacterium nucleatum and Enterococcus faecalis and their metabolites can significantly alter the expression of genes related to osteoblastic and odontogenic differentiation in SCAPs. In particular, E. faecalis supernatant suppressed cell proliferation and reduced the differentiation potential of SCAPs. In contrast, F. nucleatum increased immune and inflammatory responses in these cells. Up- or down-regulation of genes such as VEGFA, Runx2, TBX3, and NFIL3 in the presence of these bacteria can hinder the success of regenerative endodontic treatments. Therefore, the role of microorganisms in the treatment environment should be carefully considered in the design of stem cell-based therapies.

Ying et al. [9] demonstrated using animal models that advanced glycation end products (AGEs) impair osteoblastic differentiation of periodontal ligament stem cells (PDLSCs) under orthodontic forces by affecting the KDM6B/Wnt pathway. AGEs impair osteogenesis by reducing the mechanical responses of KDM6B and significantly reduce the antioxidant capacity of these cells. The study also discovered that AGEs have negative effects on the positive feedback pathway of KDM6B and Wnt, which could be used as a target for improving orthodontic treatments in diabetic patients.

This body of evidence suggests that multiple factors independently or synergistically affect the differentiation pathway of dental stem cells. Targeted use of these agents could have potential applications in the regeneration of dental tissues, treatment of genetic diseases, and improvement of endodontic and orthodontic treatments.

## Conclusion

Current evidence suggests that physical, chemical, microbial, and molecular factors directly or through signaling pathways influence the differentiation and function of dental stem cells. A detailed understanding of these effects could lead to the development of personalized therapeutic approaches in the field of restorative dentistry. Utilizing these new findings will pave the way for improved endodontic and orthodontic treatments, especially in pathological conditions such as diabetes or chronic inflammation.

## Conflict of Interest

None.
